# Development and Evaluation of a Persian‐Language Self‐Management Mobile Application for People With Spinal Cord Injury

**DOI:** 10.1111/hex.70454

**Published:** 2025-10-09

**Authors:** Amir Hossein Daeechini, Azamossadat Hosseini, Reza Rabiei, Saeed Oraee‐Yazdani, Somayeh Paydar

**Affiliations:** ^1^ Department of Health Information Management and Medical Informatics, School of Allied Medical Sciences Tehran University of Medical Sciences Tehran Iran; ^2^ Department of Health Information Technology and Management, School of Allied Medical Sciences Shahid Beheshti University of Medical Sciences Tehran Iran; ^3^ Shohada Tajrish Neurosurgical Center of Excellence, Functional Neurosurgery Research Center, Shohada Tajrish Hospital Shahid Beheshti University of Medical Sciences Tehran Iran; ^4^ Department of Health Information Technology, School of Allied Medical Sciences Kermanshah University of Medical Sciences Kermanshah Iran

**Keywords:** mobile health, self‐management, spinal cord injury, usability evaluation

## Abstract

**Introduction:**

Spinal cord injury (SCI) is associated with complex complications and long‐term disability. Mobile health applications are crucial for managing their complications and challenges, given their high adoption rate among these patients. While previous studies have developed and evaluated self‐management mobile applications for SCI in other languages and contexts, to date, no Persian‐language application tailored to the unique needs of individuals with SCI in Iran and other Persian‐speaking countries has been published. This study aims to develop and evaluate the short‐term usability of a self‐management mobile application through the participation of 20 people with spinal cord injuries.

**Methods:**

This applied developmental study was conducted in 2024 in two phases: the design and development phase and the usability evaluation phase. The application's key requirements and required capabilities were identified in the first phase, and a conceptual model was developed using Microsoft Visio. In the second phase, the application's usability was evaluated using the MAUQ questionnaire.

**Results:**

The application's menu services included modules for autonomic dysreflexia, skin care, bowel management, lifestyle, bladder management and a specialist finder. In the usability evaluation phase of the SCI self‐management mobile application, its usability score was 78.7%, signifying an acceptable level of usability.

**Conclusion:**

In this study, the design and development of the application were tailored to the needs of Persian‐speaking participants with SCI to ensure alignment with both the linguistic/cultural requirements and self‐management needs of this population. Accordingly, usability evaluation was conducted to identify and address potential issues, ensuring that the final product is comprehensive and effectively covers most complications and challenges faced by individuals with SCI.

## 1 Introduction

Spinal cord injury (SCI) is associated with complex multisystem and rare disorders, leading to degrees of voluntary muscle activity loss, sensory deprivation, and autonomic dysfunction, depending on the location and severity of the spinal cord damage [1]. SCI is one of the leading causes of mortality and disability worldwide [2].

SCI imposes a significant economic and social burden on affected individuals and their families [3], and it is estimated that SCI has a lifelong economic impact of $2–$4 billion on the global economy [4, 5]. A catastrophic health event, SCI commonly produces multiple severe comorbidities and persistent loss of bodily functions [6]. Autonomic dysreflexia, urinary tract dysfunction and infections, sexual dysfunction, pressure ulcers, depression, and gastrointestinal disorders are among the most common secondary complications and challenges faced by individuals with SCI. Ineffective management of these complications can lead to substantial physical and psychological consequences, impose significant financial burdens, and drastically alter patients' lifestyles. According to statistics, 36% of people with SCI are hospitalised at least once due to secondary complications related to the injury [3, 7, 8, 9]. Self‐management strategies provide an effective approach for reducing the incidence of secondary complications associated with SCI.

Self‐management is broadly defined as a dynamic and ongoing process through which people with chronic conditions acquire the skills and confidence necessary to manage not only their symptoms and treatments, but also the physical, psychological and social consequences of their illness. This definition emphasises behaviour change, self‐efficacy, shared decision‐making, goal setting and the development of problem‐solving skills in a collaborative patient–provider partnership. This approach is based on social cognitive theory, which states that self‐efficacy, as an individual's belief in their ability to successfully perform specific behaviours, plays a key role in the success of self‐management. In SCI, self‐management programmes aim to empower individuals to actively participate in their own care, adapt their lifestyle, and improve health outcomes by enhancing their independence and participation in health‐related decisions [10, 11, 12, 13, 14].

Smartphones have become an essential means of communication in daily life for people worldwide [15]. Mobile technologies provide effective tools for rapid communication and facilitate the transfer of demographic information, clinical data and research findings to healthcare providers [16].

Iran has a large and growing mobile technology infrastructure, which provides a suitable platform for the development of mobile health (mHealth) interventions. According to Datareportal, in 2025, the number of mobile subscriptions in Iran reached about 152 million, equivalent to 166% of the total population, and 93% of these subscriptions are connected to mobile broadband networks (including 3G, 4G and 5G). This high adoption of technology and strong communication infrastructure has provided significant opportunities for the development and implementation of mobile technology‐based interventions in the Iranian health system [17].

One such technology is mobile health applications (M‐health) [18]. mHealth applications have been developed to facilitate self‐management skills among people with various chronic conditions [7]. These applications effectively provide optimal support to people with disabilities, enabling them to take control of their health management. The primary goal of M‐health applications is to assist patients in managing their health status and delivering necessary healthcare services when in‐person consultations are not feasible [19]. Due to their high adoption rate among patients with SCI, M‐health applications are a suitable option for this patient population [3]. Research has shown that healthcare interventions based on M‐health applications enhance patients' self‐efficacy, prevent complications, improve quality of life and reduce the frequency of hospital readmissions [20, 21, 22].

## 2 Significance of the Study

The necessity of education, awareness promotion, and the development of supportive programmes for patients with SCI, along with the need to manage complications and symptoms associated with SCI, makes these patients suitable candidates for the design, implementation, and evaluation of a mobile application aimed at supporting self‐management. Developing a self‐management mobile application for patients with SCI may facilitate better self‐management for individuals with SCI by providing accessible, user‐friendly tools for managing common complications. Innovative technologies, such as mobile health applications, can be facilitative and supportive for patients and healthcare providers. Mortenson et al. [23] described the development of a self‐management app for people with SCI in Canada, utilising a participatory, user‐centred process and preliminary field‐testing. More recently, Middleton et al. [24] co‐designed and evaluated the Spinal Cord Injury Health Maintenance Tool with people living with SCI and multidisciplinary stakeholders in Australia, demonstrating the feasibility and acceptability of using mHealth tools to support key aspects of SCI self‐management.

Despite the growing number of mobile health (mHealth) applications developed for people with SCI, several important gaps remain in the literature. First, almost all published SCI self‐management apps have focused on English‐speaking populations. To date, there is no published mobile app specifically developed and validated for Persian‐speaking individuals with SCI, a significant population in Iran and other Farsi‐speaking countries. This indicates that mobile health options tailored to the linguistic, cultural, and healthcare contexts of Persian‐speaking populations (including Iran, Afghanistan, and Tajikistan) do not exist. Second, many existing mHealth interventions are based on top‐down designs, with limited direct involvement of end users in the needs assessment, feature definition, and iterative evaluation stages, leading to potential mismatches between app functions and user priorities. Moreover, a considerable proportion of published SCI self‐management apps focus on a single complication, such as pressure ulcer prevention [25], serious games [26], home‐based exercise [27], and rehabilitation [7].

Also, in Iran, self‐management support for individuals with SCI remains largely limited to episodic hospital‐based rehabilitation services, occasional educational workshops and traditional printed materials. Such approaches are often poorly accessible to those in rural or underserved regions, with ongoing follow‐up and structured programmes generally unavailable. There are no established nationwide digital or mobile‐based self‐management programmes for SCI patients. This lack of accessible, linguistically and culturally appropriate, and interactive self‐management support underscores a major service gap, particularly given the widespread adoption of smartphones among Iranian adults. The development of a dedicated mobile application co‐designed with SCI patients to ensure relevance to their needs therefore, represents a critical opportunity to address unmet needs in the Iranian context by expanding the reach, continuity, and personalisation of self‐management support. Accordingly, the study objective is to fill these gaps by developing and evaluating a self‐management mobile application for individuals with SCI in Iran and supporting them in coping with SCI and improving their quality of life. The development and evaluation of the SCI self‐management mobile application in the present study was based on several valid behavioural science and digital health frameworks. The content shaping and determination of the required features of the application were based on the self‐management support model [12]. This model focuses on self‐efficacy and changing user behaviour. The application development was based on a person‐centred approach so that the content used in the app was adjusted based on the needs and preferences of Persian‐speaking users [28]. This will improve usability and user satisfaction. In addition, in the evaluation phase, the valid Mobile Application Usability Questionnaire (MAUQ) [29] tool, which is based on up‐to‐date digital health frameworks, was used to assess usability to enable a comprehensive evaluation [30].

## 3 Methods

This applied developmental study was conducted in 2024 in two phases, including the design and development phase of the application and the usability evaluation phase of the SCI self‐management mobile application.

Participants were purposively selected from the SCI outpatient clinic and patient support associations affiliated with Shahid Beheshti University of Medical Sciences. Recruitment was conducted via clinic referrals and targeted invitations through patient networks. Interested individuals received detailed information about the study and provided informed consent to participate. No financial compensation or incentives were provided to participants for their involvement in the study. Participation was entirely voluntary. We aimed for a sample size of 20 participants, which aligns with usability testing guidelines suggesting that 15–20 users can identify the majority of usability issues in digital health applications [31]. This sample size is commonly employed in early‐phase app usability evaluations to yield sufficient feedback while maintaining feasibility given the targeted, specific user group.

**Inclusion criteria:**

- Age 18 years or older.
- Diagnosis of SCI (traumatic or non‐traumatic) confirmed by a physician.
- Ownership and regular use of a smartphone running.
- Ability to read and communicate in Persian.
- Willingness and ability to use the app for a minimum of 2 weeks and complete the usability questionnaire.

**Exclusion criteria:**

- Cognitive impairments preventing independent app use.
- Significant visual or upper‐limb motor impairments that would preclude effective use of a smartphone.

### 3.1 First Phase: Mobile Application Design and Development

A comprehensive literature review was performed in Scopus, PubMed, Web of Science, and Google search engines using the following keywords: SCI, mobile health, self‐management, and requirement. Studies were included if they were original articles, reviews, guidelines, or scientific reports published in English until July 2024. Eligible sources focused on mHealth interventions, applications, or digital tools developed for self‐management among individuals with SCI, and provided information on app requirements, design features, user needs, usability, or user‐centred development processes. Studies were excluded if they did not focus on mHealth or digital interventions for SCI, included only non‐SCI populations, were commentary articles, editorials, or conference abstracts lacking primary data or detailed app requirements, or were published in a non‐English language without accessible translation. All potentially relevant abstracts and full texts were independently screened by two researchers. Disagreements were resolved by discussion or consultation with a third reviewer. After analysing the retrieved studies, the key requirements and required capabilities were extracted into an Excel file. The requirements and app functionalities were mapped to key elements of the Self‐Management Support Model and validated against digital health design principles to ensure both evidence‐based content and optimal user engagement. Subsequently, a researcher‐developed questionnaire was designed based on a 5‐point Likert scale.

After expert and preliminary patient review, the application needs assessment incorporated iterative, participatory elements: initial findings and prototypes were shared with a panel of SCI patients, and their comments were used to further refine functional priorities and content. Users played a role in the various stages of app development and evaluation beyond simply rating predefined features and completing the MAUQ. The development process involved spinal cord user participation and feedback at several stages: (1) direct rating and prioritisation of requirements via questionnaire, (2) review and discussion of preliminary wireframes and screens in individual sessions, and (3) collection of structured feedback including through the application's “Contact Us” function to inform further modifications before final usability evaluation. The conceptual model of the application was thus shaped through both expert and end‐user input, following principles of participatory, user‐centred design as far as structurally possible.

The questionnaire consisted of three sections: demographic information (10 items), key requirements (24 items), and required capabilities (9 items) of the self‐management mobile application. The content validity of the questionnaire was approved by an expert panel comprising five neurosurgeons, five physiotherapists, five health information management specialists, and five medical informatics specialists, who were university faculty members with 10 years of relevant experience. Neurosurgeon and physiotherapist experts were required to have substantial direct experience in the care, rehabilitation, or management of patients with SCI, including involvement in clinical treatment, research, or educational programmes related to SCI. Health information management and medical informatics experts were required to demonstrate significant experience (minimum 10 years) in medical app development, or research involving mHealth solutions for chronic or disabling conditions, including SCI, where possible.

The questionnaire's reliability was checked, and the internal consistency of the items was calculated (Cronbach's *α* = 0.94). The validated questionnaire was then distributed to five physiotherapists, five neurosurgical nurses, 10 neurosurgeons, and separately to 20 patients with SCI to rate the importance of each questionnaire item on a scale of 1–5, with 5 indicating the highest level of significance and 1 indicating the lowest. The samples of experts were recruited using purposive sampling. Eligible experts were identified from staff lists at Shahid Beheshti University of Medical Sciences, focusing on those with a minimum of 5 years' direct clinical experience with SCI patients. The 20 individuals with SCI were recruited through the SCI outpatient clinic using clinic referrals. Based on the findings from the needs assessment, a conceptual model of the application, including functional, structural, and behavioural models, was designed using Microsoft Visio 2021.

The conceptual model of the application was validated by a panel of five medical informatics specialists and five health information management specialists. The panel members were purposively selected based on their extensive experience. Each had at least 10 years of academic and professional experience in relevant fields. Many of the panel members were academics who were actively involved in research and development of mobile health technologies and chronic disease management. A prototype of the application was coded by a team of experienced software engineers, following the approved conceptual model and user requirements. In the next stage, specialists conducted testing and debugging processes to ensure functionality and performance optimisation.

### 3.2 Second Phase: Usability Evaluation of the Mobile Application

In this phase, the usability evaluation of the mobile application was conducted with the participation of patients with SCI. The usability evaluation by the standardised MAUQ questionnaire, completed by all participants after a minimum of 2 weeks' use. The study did not include additional triangulated data sources, such as in‐depth interviews, focus groups, digital usage analytics, or direct observational methods. This approach provided valuable initial quantitative usability data but limited deep qualitative insight and behavioural usage patterns. The MAUQ was used as the assessment tool. This questionnaire was developed by Zhou et al. [29] to evaluate the usability of mobile health applications. The MAUQ questionnaire consists of 18 items categorised into three sections: ease of Use (5 items), interface and user Satisfaction (7 items), and Usefulness (6 items).

The MAUQ is designed based on a 7‐point Likert scale, ranging from 'completely disagree' (1 point) to 'completely agree' (7 points). To create the Persian version, the original English MAUQ was forward‐translated independently by three expert translators familiar with medical terminology and digital health instruments. The translations were synthesised into a single draft by consensus. This version was then back‐translated into English by an independent bilingual translator not previously involved in the study. Discrepancies between the back‐translation and the original were discussed and resolved to ensure conceptual equivalence. The preliminary Persian draft underwent face and content validity assessment by a panel of five medical informatics and five health information management specialists. Where necessary, minor adjustments were made for cultural and linguistic clarity relevant to the Iranian context. Its reliability had already been confirmed in previous studies. For data analysis, the obtained questionnaire scores were classified into three levels. The application was provided to 20 patients with SCI who had varying levels of impairment and owned a smartphone. They were instructed to use the application for a minimum of 2 weeks. During the usability phase, participants were encouraged to submit feedback and suggestions via the app's ‘Contact Us’ feature. All submitted feedback was periodically monitored, collected, and anonymized by the research team. The content of user feedback was systematically reviewed and qualitatively analysed to identify potential usability problems or suggestions for improvement. These insights directly contributed to refining the application's features and user interface.

Following this period, the MAUQ usability questionnaire was distributed among the participants. The collected data were analysed using SPSS version 26.

Descriptive statistics, including means, standard deviations, frequencies, and percentages, were used to summarise participants' demographic characteristics, overall and domain‐specific usability scores (MAUQ), and the importance ratings for app requirements and features.

### 3.3 Ethical Considerations

This article is a part of the master's thesis project titled 'Development of a Mobile Application for Self‐management of Spinal Cord Injury Patients', approved in 2024, which was reviewed and approved by the review board and ethics committee of Shahid Beheshti University of Medical Science. Ethics Approval Code: IR.SBMU.RETECH.REC.1402.858. The ethical and reporting components of this study were aligned with recognised digital health research guidelines, including the mERA checklist, to ensure transparency in development, evaluation, and data handling. All participants received a clear explanation of the study's purpose, procedures, and their expectations before enrolment. informed consent was obtained from all participants before their involvement in any part of the research. Regarding user feedback collection, participants were informed that any suggestions, comments, or communication they provided via the app's 'Contact Us' feature would be collected for research purposes but would be fully deidentified and analysed in aggregate. No personal identifiers or sensitive information submitted through the feedback function were published or disclosed. Participants were assured of their right to withdraw at any time without prejudice. All screenshots display interface elements only, without depicting any individual's identifiable information. Inclusion of these images in publications was addressed in the participant informed consent process.

## 4 Results

### 4.1 Findings from the Design and Development of the SCI Self‐Management Mobile Application

The findings from the development phase indicate that the identification of key components of the application was a seamless and multi‐stage process. First, these components were extracted through a systematic review of information databases and guidelines related to SCI self‐management based on the established criteria. In this phase, components that were mentioned in more than 20% of the articles and guidelines were listed and selected. Then, these key components were reviewed and prioritised in meetings with experts including neurosurgeons, physiotherapists, health information management specialists, and medical informatics specialists. In these meetings, the experts assessed and prioritised these key components or requirements based on treatment priorities, self‐management, and cultural contexts. The key components that had gained agreement from more than 75% of the experts were considered in the design of the application. Then, in the next stages, these components were needs assessed by patients and experts in the form of a questionnaire (as presented in Section Results, 3).

**Figure 1:**
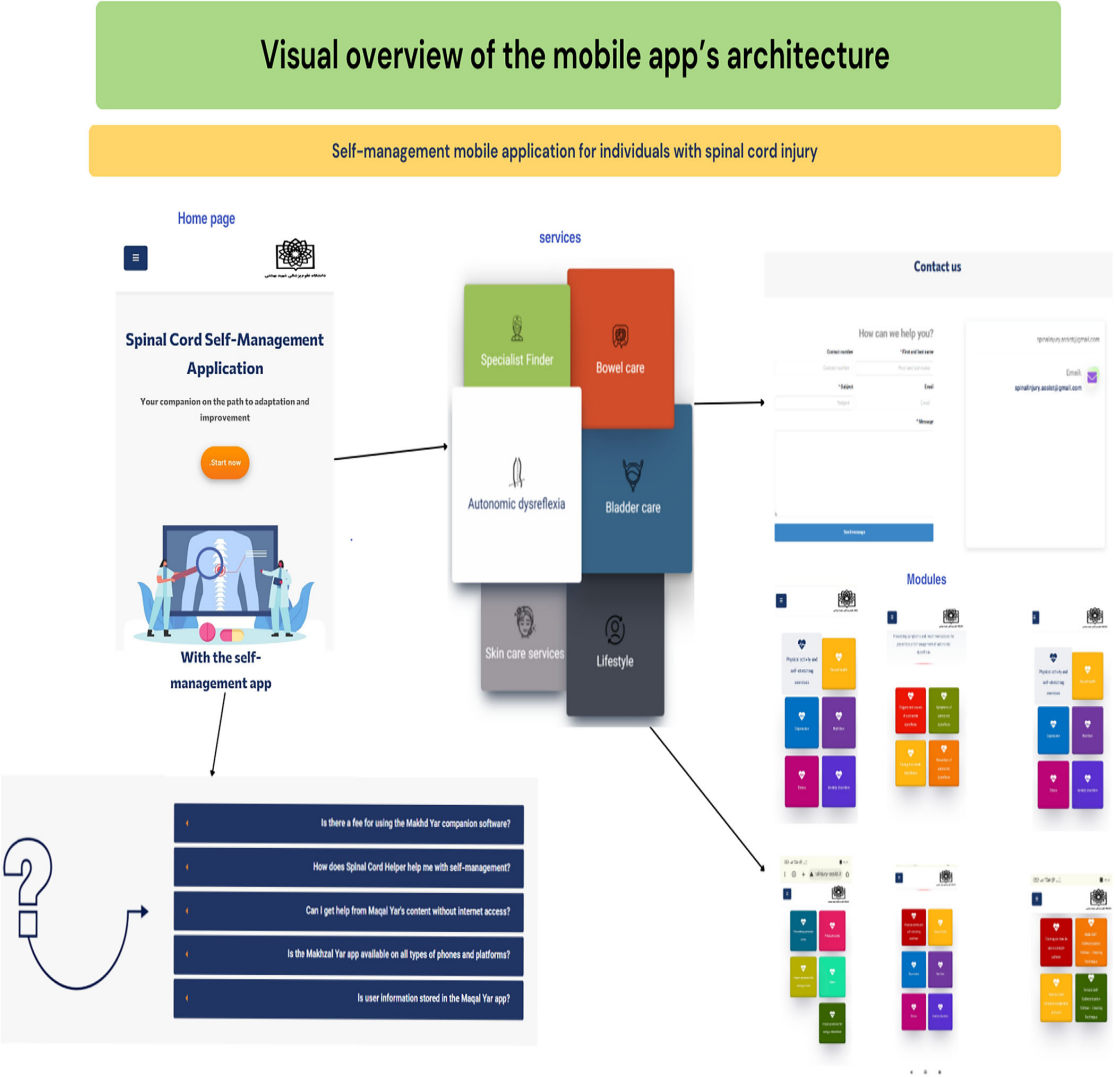
Visual overview of the mobile apps'architecture.

A schematic of the overall architecture, navigation logic, and how to access the different sections of the mobile app is shown in Figure 1. This schematic helps to increase understanding of the design and flow of the application. This schematic diagram shows the overall structure of the app, showing the six main modules, the home page, the FAQ section, and the Contact Us section. This schematic diagram helps to increase understanding of the design and flow of the app. This structure enables users to seamlessly navigate through the different educational and self‐management sections, ensuring comprehensive coverage of the most important areas of SCI care. The navigation paths are arranged in such a way that the user's interaction with the app is smooth and continuous, and no important part of the educational content or functionality is left out of the user's view. This figure provides a summary of the main modules and navigation paths of the SCI self‐management app.

**Figure 2:**
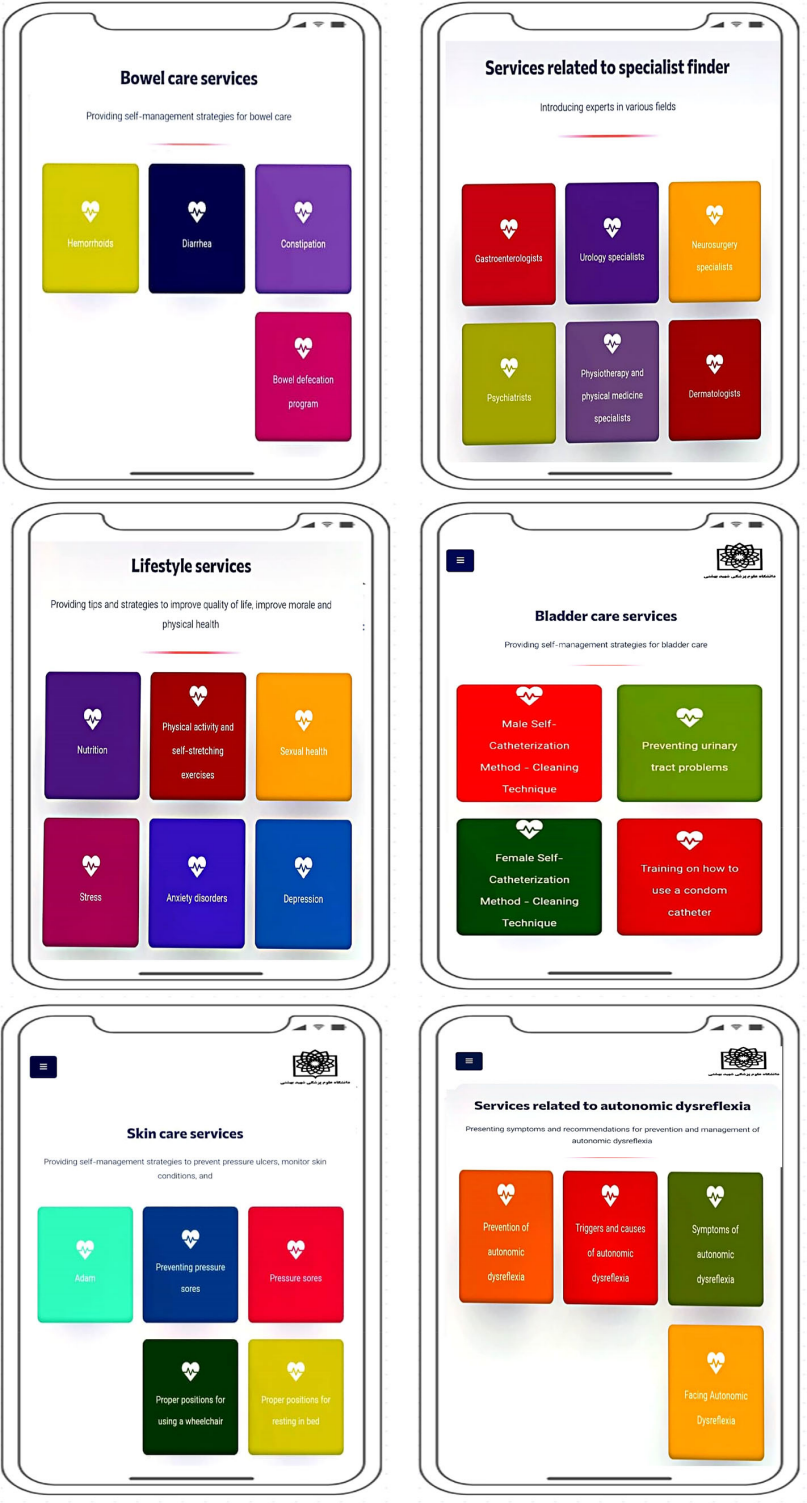
Wireframe of spinal cord injury self‐management mobile application modules.

Figure 2 shows a wireframe of the SCI self‐management app modules. The six main modules of the app include the modules for bowel care, bladder care, skin care, autonomic dysreflexia, lifestyle and specialist locator. Each of these modules contains several sub‐modules that provide comprehensive information and practical training. The content of each module is designed to help SCI patients self‐manage, providing practical and supportive solutions to help improve their quality of life and better manage their medical conditions.

Key findings from the literature review are presented in Table 1. Two studies were from Canada, and one study was from the United States, Australia, Italy, and Switzerland. All of these applications were in English. They were developed on IOS or Android platforms. Except for the study by MacGillivray et al. [7] the other studies explicitly stated the technical features of the developed applications.

**Table 1:** Key findings from the literature review.

ID	Study author (year)	Country	Study type	Platform	aim	Number of participants	Technical features	App content
**1**	Mortenson et al. [23], (2019)	Canada	A qualitative descriptive approach	IOS	Development of a functional mobile app to facilitate self‐management skills needed to prevent secondary complications following recent SCI during inpatient rehabilitation.	75	Medication tracker/appointment calendar/Daily mood journal/symptom tracker/Daily vitals	SCI goal‐setting tool, bowel and bladder, spasticity/nutritional planner, and recreational resources
**2**	Middleton et al. [24], (2024)	Australia	Mixed methods codesign	NM	Describes the collaborative design process for the development of the Spinal Cord Injury Health Maintenance Tool (SCI‐HMT),	Interviews: 18 end‐user testing: 41	Screening tools (quick health checker)/symptom trackers/diaries, goal‐setting programmes/collaborative care plans/toolboxes of self‐management strategies (for bowel, mental health, pain, and pressure injury)/quick self‐management and prevention tips/and prompts (red flags) for when to seek further medical attention and advice at the right place and time.	Bladder, bowel, skin, pain, and autonomic dysreflexia, mental health
**3**	Amann et al. [25], (2020)	Switzerland	Mixed methods study (codesign approach + usability testing)	Android	Develop an evidence‐based, self‐management app to support individuals with SCI in the prevention of pressure injuries	15 + 5	Smart camera/pressure injury diary/expert consultation/reminder/knowledge repository	Support surface, repositioning, nutrition, skincare, skin assessment, exercising, collaboration with health professionals/caregivers, transfers, clothing, body function and structure, personal factors, and general
**4**	Bizzarini et al. [27], (2022)	Italy	Pilot study	Android	Evaluates its feasibility with a pilot study in a real clinical intervention	14	Video, text, and picture instructions/game‐like features	Exercise programme
**5**	Meade and Maslowski [26], (2018)	USA	Mixed Methods (development+feasibility evaluation)	Android/IOS	Development & evaluation of SCI Serious Gaming promotes self‐management	14	Game/customising/health metrics	Bowel and bladder/stress/fitness/energy fatigue/cognitive flexibility; resilience/skin
**6**	MacGillivray et al. [7], (2018)	Canada	Pilot feasibility study	Android	Feasibility of the study to evaluate a self‐management mobile app	17	NM	Bowel (tracker/journal), bladder (tracker/journal), skin, autonomic dysreflexia/orthostatic hypotension, urinary tract infections, spasticity, physical activity, SCI goals, SCI confidence, pain, fluid intake (tracker/journal), fatigue, equipment, and neurological status

Abbreviation: NM, not mentioned.

**Figure 3:**
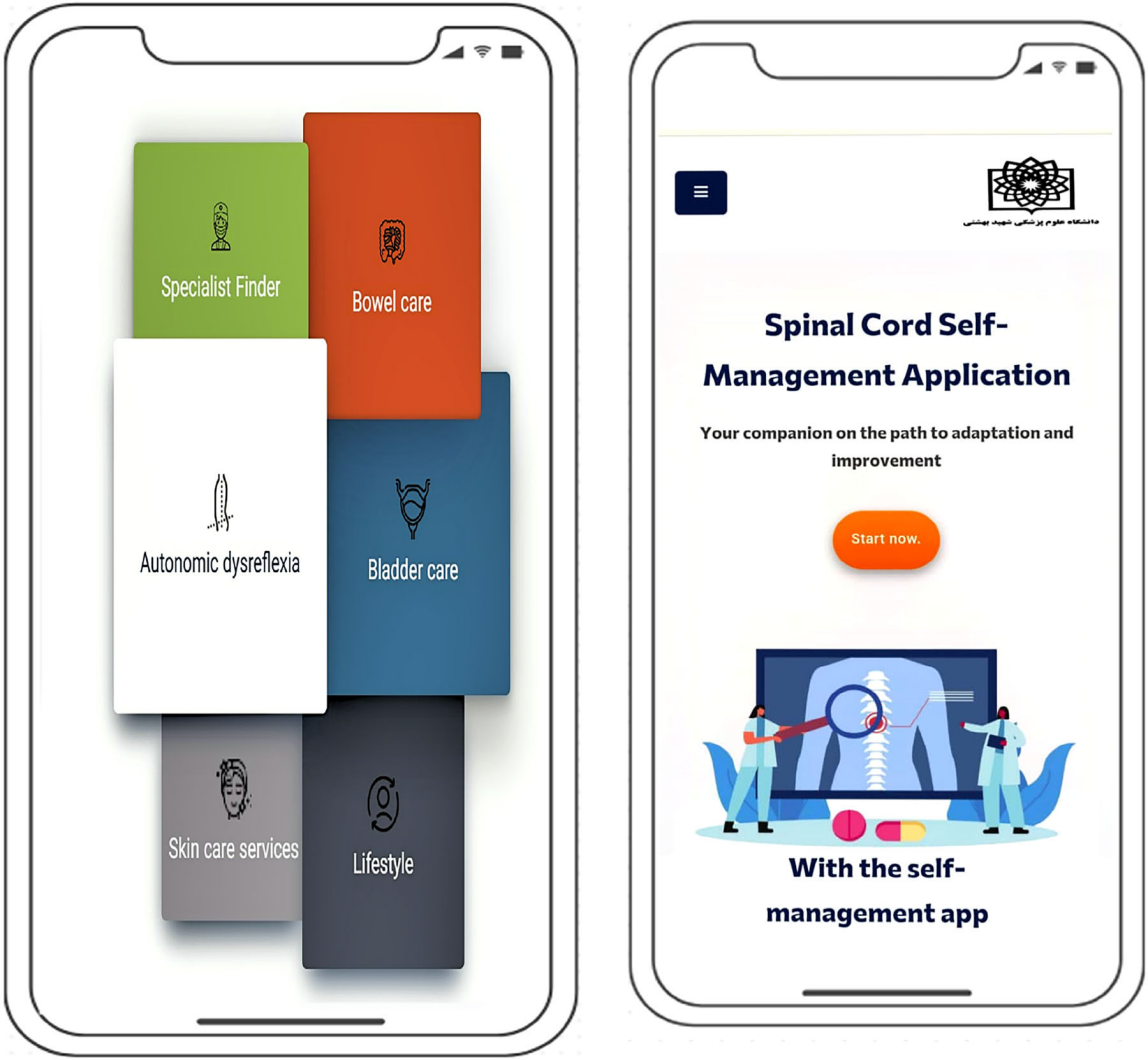
Screenshot of the menu and home page of the self‐management mobile application.

The SCI Self‐Management App Home Page and App Menu Services are shown in Figure 3. This figure depicts a view of the SCI Self‐Management App Home Page and Menu, which is the user's first point of contact with the app. On this page, the main menu contains six key app modules: Autonomic Dysreflexia, Skin Care, Bowel Care, Lifestyle, Bladder Care and Find a Specialist. This section is designed to provide quick and easy access to all modules so that users can navigate the different sections of the app easily and without confusion, depending on their needs. The visual layout of the app menu and home page is such that even users with different levels of disability can use it easily. This figure well emphasises how user interface design helps to enhance the smooth and functional user experience and guides users on the path to effective SCI self‐management. The visual layout, structure, and user‐friendly accessibility of the app are designed to support intuitive navigation between sections for people with varying levels of impairment. Thus, Figure 2 not only shows the visual appearance of the app's home screen and menu, but also highlights the importance of user‐centred and accessible design in the app's success.

Table 2 lists the modules of the SCI mobile application along with its sub‐modules. This table shows the structured framework and division of the application modules.

**Table 2:** Summary of core contents by module.

Module	Submodules	Description
Skin care	Oedema	Strategies for preventing and managing wounds
	Appropriate positions for sleeping	
	Pressure ulcers	
	Prevention of pressure ulcers	
	Suitable positions for using a wheelchair	
Autonomic dysreflexia	Symptoms of autonomic dysreflexia	Information on controlling of autonomic dysreflexia episodes
	Triggers and causes of autonomic dysreflexia	
	Prevention of autonomic dysreflexia	
	Dealing with autonomic dysreflexia	
Bowel management	Diarrhoea/constipation/haemorrhoids/bowel schedule	Strategies for bowel control and care
Bladder management	Self‐catheterisation method for men	Education on bladder management techniques
	Self‐catheterisation method for women	
	Training in using a condom catheter	
	Training in cleaning urination equipment at home	
	Prevention of urinary tract problems	
Lifestyle	Physical activity and self‐stretching/exercises/nutrition/depression/stress/anxiety disorders/sexual health	Recommendations to improve overall health
Specialist finder	Gastroenterologist/urologist/neurosurgeon	Introducing some of the best specialists related to spinal cord injury in the form of a specialist finder
	Skin specialist/physiotherapy specialist psychiatrist	

**Figure 4:**
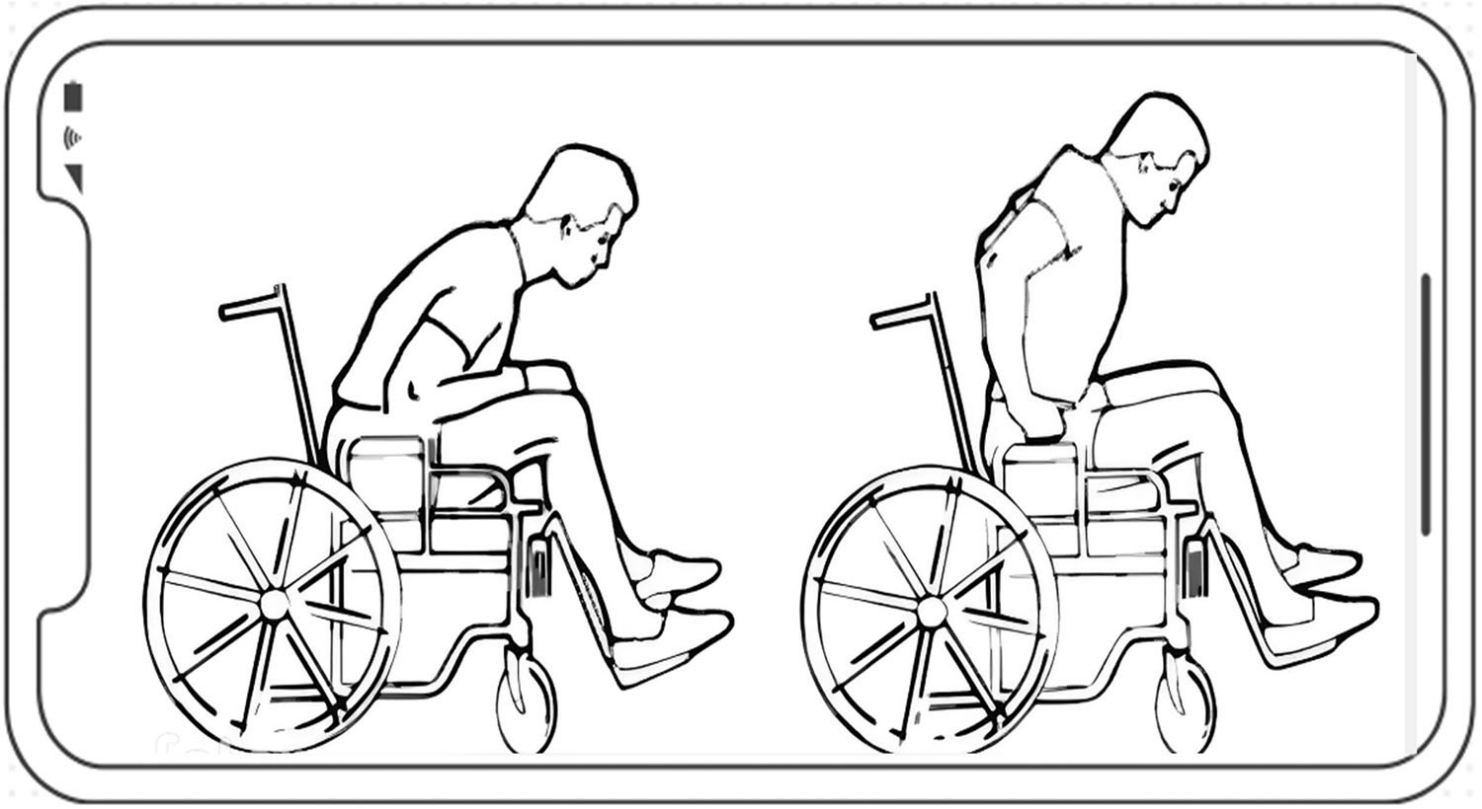
Application image gallery page.

Figure 4 shows one of the images used in the 'Image Gallery' section. The Image Gallery section presents visual educational content that demonstrates self‐management techniques and strategies in a visual and understandable way. These images help users who may have a lower level of text literacy or prefer visual learning to better understand the concepts and benefit from the application more effectively. The combination of text and image content in these sections provides a multimedia approach to education that increases engagement and better retention of information. For example, the image shown in the image above is one of the cases of correct wheelchair use training, which aims to prevent pressure ulcers in SCI patients. This figure shows how visual content is integrated into the application to enhance learning and support users with different literacy levels. This user‐centred design ensures wider usability and greater impact of the application.

**Figure 5:**
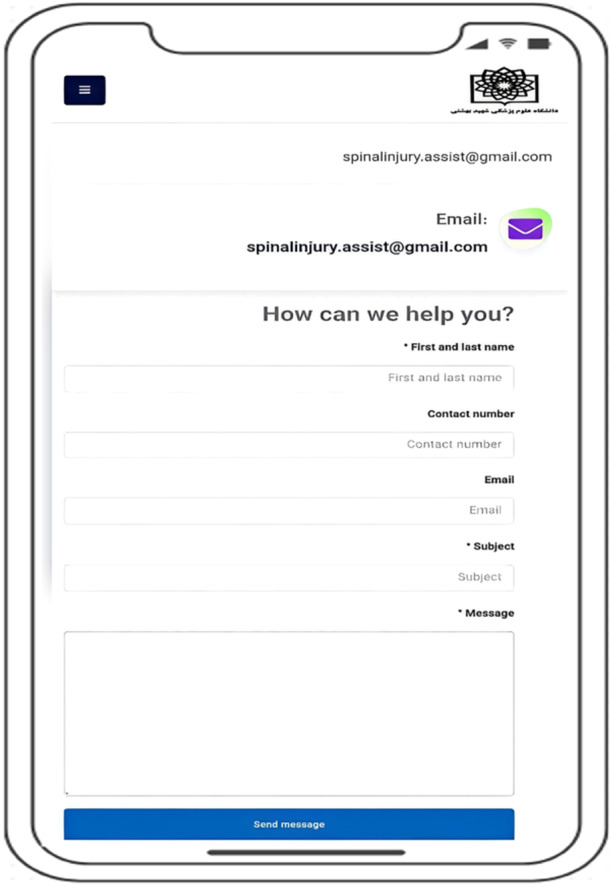
Contact page for mobile application developers.

Figure 5 shows a view of the 'Contact Us' section of the SCI self‐management app, which is designed as a direct channel between users and the development team. This section is included to improve the app after its development and to incorporate feedback and suggestions from participants with SCI. SCI users and others working with the app can access this section to communicate directly with the SCI self‐management app developers. By facilitating ongoing user input, this feature ensures that the app remains responsive to patient needs and can be iteratively improved based on real‐world user experiences. This feature creates an effective, two‐way interaction that helps to continuously improve the app. In this way, the app evolves dynamically and user‐centred, not only in the early stages of development but also throughout its lifespan. This approach ensures that the services provided are constantly improved in line with changing patient needs and current standards. Also, this section increases the sense of participation and support of users in the development process, which in turn improves their satisfaction and loyalty to the application.

### 4.2 Findings from the Usability Evaluation of the SCI Self‐Management Mobile Application

Following the development of the application, the SCI self‐management mobile application was provided to users for evaluation. To assess usability, the application was made available to 20 users with SCI at varying levels of severity. One of the researchers patiently and clearly explained the study's objectives to the participants. All users participated in the evaluation process and completed the questionnaire. The overall mean usability score reported by the participants was 5.51, which falls within an acceptable range. Among the three usability evaluation criteria, ease of use received the highest average score of 6.09. The user interface and satisfaction component scored 5.71, while the application's usefulness had an average score of 4.73. Notably, the “ease of use” subscale of the MAUQ received the highest average score (6.09 out of 7), while the “usefulness” domain received a lower mean score (4.73 out of 7). This indicates that although participants found the application simple and intuitive to navigate, some were less convinced of its practical impact in managing their SCI‐related needs.

It should be noted that usability findings reflect only participant self‐reported experiences as measured by the MAUQ following short‐term app use. Objective engagement metrics, such as the total time spent in the app, the number of sessions, or the frequency of accessing each module, were not collected. Consequently, we are unable to report which modules were most frequently accessed or whether levels of engagement correlated with perceived usability or usefulness. The participants' demographic data in the usability evaluation and the detailed findings of user scores for the SCI self‐management application are presented in Tables 3 and 4.

**Table 3:** Demographic information of evaluators.

Index	Variables		Number (*n*)	Frequency (%)	Variability metrics
1	Sex	Male	15	75	—
		Female	5	25	
2	Material status	Single	12	60	—
		Married	8	40	
3	Age (years)	20–30	8	40	Mean: 34.5 ± 9.2 (SD)
		30–40	9	45	
		40–50	2	10	
		50–60	1	5	Range: 20–60
		60<	0	0	
4	Level of education	High school	1	5	—
		Diploma	6	30	
		Associate	4	20	
		Degree	5	25	
		BSc	3	15	
		MSc	1	5	
		PhD			
5	Time since injury	Less than a year	6	30	Median: 3 years (IQR: 1–8)
		1–5 years	9	45	
		5–10 years	3	15	Range: < 1 to > 10 years
		More than 10 years	2	10	
6	Prior experience with smartphones (years of use)	1–5 years	1	5	Mean: 9.2 ± 4.1 (SD)
		5–10 years	4	20	
		More than 10 years	15	75	Range: 1–> 10 year
7	Type of injury	Complete SCI	5	25	—
		Incomplete SCI	15	75	
**8**	**Total**		**20**	**100**	—

Abbreviations: SCI, spinal cord injury; SD, standard deviation.

**Table 4:** Frequency distribution and scores of users participating in the usability evaluation of the spinal cord injury application.

Mean (%)	Mean (score)	Max (score)	Min (score)	Number (*n*)	Items
87	6.09	7	2.8	20	Ease of use
81.6	5.71	7	1	20	User interface & satisfaction
67.6	4.73	7	1	20	Usefulness
78.7	5.51	20			Total

To examine the effect of demographic variables in Table 1 (gender, age, education level, type of injury, etc.), a subgroup analysis of these variables was conducted. The results showed that subgroups of these variables did not have a significant effect on the overall usability score. For example, no statistically significant differences were observed between male and female participants in the overall usability scores or any of the MAUQ subdomains (ease of use, user satisfaction and usefulness). This finding is consistent with previous studies in mHealth usability, which report minimal or no gender effects on perceived usability [32]. However, due to the limited sample size in the present study, the results of this analysis cannot be generalised to larger populations and may differ in larger samples or other studies. For this purpose, it is suggested that more comprehensive studies be conducted in larger samples in future studies.

## 5 Discussion

The SCI self‐management mobile application was designed to provide educational information on key self‐management strategies related to skin care, autonomic dysreflexia, bowel management, bladder management and lifestyle, with the aim of supporting individuals with SCI in Iran and supporting them in coping with SCI and improving their quality of life. Feedback collected during usability evaluation suggested that users found the application helpful in addressing some of these areas, although further evaluation may be needed to confirm its comprehensiveness.

In this study, the development and evaluation phases, the principles of user participation and the use of iterative feedback were observed following the principles of the Person‐Based Approach (PBA) [28]. In addition, the usability evaluation was conducted with the standard MAUQ questionnaire, which is part of the standard evaluations in the field of digital health. However, the mERA framework [30] was also considered as a broad guide to clarify the required elements as part of the development phase.

An online survey conducted in Canada (2014) revealed that nearly 75% of people with SCI considered the development of self‐management programmes for individuals with SCI to be highly essential [33]. Developing a self‐management mobile application for people with SCI can be essential in increasing awareness and empowering these individuals. Emphasising innovative technologies, including mobile health applications, can be facilitative and supportive for individuals with SCI and their caregivers.

One of the key findings of the present study is the significant difference in the scores of ‘ease of use’ and ‘usefulnes’. This difference shows that having a simple and understandable user interface alone does not mean that the app is successful in responding to all aspects of the needs of SCI patients. While ‘ease of use’ focuses on the appearance and functional aspects of the app, ‘usefulness’ focuses on the practical impact of the app in helping to manage the needs of patients. This difference in scores between ‘ease of use’ and ‘usefulness’ is also found in the study by Mortezai et al. [34] and other similar studies. Since one of the important reasons for this difference in scores between ‘ease of use’ and ‘usefulness’ is due to the diversity and complexity of the needs of SCI patients, even the best mobile health tools cannot fully meet all the needs of these patients. Therefore, to increase ‘usefulness’, future studies should focus on expanding app capabilities, increasing personalisation options, and integrating user‐generated content to better cover the full spectrum of patient needs, thereby increasing perceived usefulness along with ease of use.

While several mobile applications have been developed internationally to support self‐management in individuals with SCI, the present study's approach diverges from much of the existing literature in key respects. First, unlike most published SCI apps, which predominantly target English‐speaking users and are often designed with limited input from local patients, our application is the first to be specifically tailored to the linguistic and cultural needs of Persian‐speaking users, filling a major gap for Farsi‐speaking populations in Iran and neighbouring countries. In contrast to studies such as Amann et al. [25] and Bizzarini et al. [27], which have focused on certain aspects of self‐management or single complications (e.g., pressure ulcers, Home‐Based Exercise), our studies offers a more comprehensive suite of modules, including autonomic dysreflexia, skin care, bowel and bladder management and lifestyle support. Furthermore, our user involvement extended beyond feature rating: patients provided iterative feedback throughout design and usability testing, although, similar to previous reports, some constraints limited the depth of codesign. In sum, our application is distinctive for its cultural and linguistic adaptation, relatively broad content coverage and iterative instead of purely top‐down development.

The mobile application developed in the present study adopts a more comprehensive approach compared to studies such as Amann et al. [25] and Bizzarini et al. [27] by offering a diverse menu of services and focusing on self‐management strategies. In this study, the development of the mobile application was based on a needs assessment conducted among both specialists and people with SCI. The feedback and suggestions from healthcare professionals and individuals with SCI were carefully reviewed and incorporated as much as possible. Additionally, the application was evaluated by 20 users with SCI, receiving an acceptable usability score based on the conducted assessment. According to the findings of this study, although the usability of the application was satisfactory, its effectiveness has not been proven so far. To evaluate the effectiveness of the SCI app, it is necessary to conduct studies for this purpose in future research. In addition, the acceptance of the application developed in the present study by healthcare institutions requires the examination of various factors, including the development of infrastructure and the availability of resources and budgets of those institutions. Therefore, any prediction about the effectiveness or acceptance of the SCI self‐management application is speculative and requires further investigation in future studies.

In future studies, advanced technologies such as artificial intelligence (AI) can enhance the capabilities of smart mobile applications by analysing patient data and providing personalised recommendations. This integration can contribute to improved monitoring, supervision, and management of people with spinal cord injuries [35].

## 6 Strengths and Limitations

One of the strengths of this study is the involvement of people with SCI, who are the primary users of the mobile health application, in both the needs assessment and usability evaluation phases. While user participation was central to both phases of this study, it is important to critically consider the depth of this involvement. Participants contributed not only by rating and prioritising features, but also through feedback during prototype review sessions and via the app's Contact Us function, which led to specific refinements in interface navigation and emphasised the need for culturally sensitive illustrations. Future studies could strengthen codesign by involving users more extensively throughout iterative development and evaluation cycles to further enhance the relevance and impact of the application. Another notable strength is the utilisation of insights, suggestions, and consultations from an experienced multidisciplinary team, including neurosurgeons, nurses, physiotherapists, health information technology experts, health information management specialists, and medical informatics professionals.

This study has certain limitations. The main constraint of usability evaluation was to rely on the MAUQ questionnaire after a short period of use. Study design did not include quality interviews, observational studies, or use reports (such as programme use reports, time spent at each session, and access to individual modules), which could provide a richer and more detailed insight into how people interact with the programme, identifying pain points, and offering improvement. Future studies should adopt a combined approach that includes quantitative questionnaires and user qualitative feedback as well as programme use data, to create a more comprehensive understanding of the realisation and interaction in the real world. Another limit is the sample size, as only 20 users with SCI participated in this study at Shohada Tajrish Hospital and Nasim Clinic in Tehran. This limitation limits the generalisation of the findings to the larger population. Another limit is that during the assessment of the needs and the requirements of the mobile programme, only self‐management resources, articles, and guidelines were considered in English, while resources were not included in other languages.

## 7 Conclusion

This study was successfully conducted to develop and evaluate the usability of a mobile application for self‐management of individuals with spinal cord injuries. The educational content was categorised into six domains, and the necessary components for each category were identified. By adopting a user‐centred approach, people with spinal cord injuries were involved throughout the development and evaluation phases to ensure that the mobile application aligned with their needs. Accordingly, usability evaluation was performed to identify and resolve potential issues, ensuring that the final product aligns with user needs and is accessible. The comprehensive impact on clinical outcomes or user knowledge remains to be evaluated in future studies.

## Author Contributions

**Amir Hossein Daeechini:** conceptualisation, methodology, writing – review and editing. **Azamossadat Hosseini:** supervision, conceptualisation, methodology, review and editing. **Reza Rabiei:** formal analysis, investigation. **Saeed Oraee‐Yazdani:** data gathering, investigation. **Somayeh Paydar:** formal analysis, investigation, review and editing. Data gathering, Investigation. All the authors read and approved the final manuscript.

## Ethics Statement

This article is part of the master's thesis project titled ‘Development of a Mobile Application for Self‐management of Spinal Cord Injury Patients’, which was approved in 2024. The review board and ethics committee of Shahid Beheshti University of Medical Sciences reviewed and approved it (IR.SBMU.RETECH.REC.1402.858).

## Consent

The authors have nothing to report.

## Conflicts of Interest

The authors declare no conflicts of interest.

## Data Availability

All data generated or analysed during this study are included in this published article.
